# Global citizenship online in higher education

**DOI:** 10.1007/s10671-023-09351-6

**Published:** 2023-06-01

**Authors:** Francesca Helm, Alice Baroni, Giuseppe Acconcia

**Affiliations:** Department of Political Science, Law and International Studies, University of Padova, Padua, Italy

**Keywords:** Global citizenship education, Global competence, Intercultural communication, Online dialogue, Virtual exchange

## Abstract

Discourses of global education, citizenship and competence have been characterising the higher education literature in recent years. The COVID-19 pandemic has both heightened the relevance of global citizenship education and presented new challenges as educators and students continue to grapple with the lasting impact and implications. This paper presents the findings of a research study which looked at the perceived learning outcomes of a ‘virtual exchange’ project which addressed issues relevant to global citizenship, involving students in European and Southern Mediterranean countries in online dialogue programmes. The study used quantitative and qualitative approaches to the analysis of responses to open survey questions using the quantitative tool IRAMUTEQ (Sbalchiero & Tuzzi, 2016) and focus groups. Participants perceived that their learning was happening above all through their encounters and discussions with people from different backgrounds. They reported learning to listen actively and carefully, to accept and/or respect different opinions and experiences. The findings open up possibilities for how higher education institutions might engage students in online transnational and global learning experiences—which can contribute to thinking about renewing education and societies in a post-pandemic world.

## Introduction

While the concept of international education is not new, this century has seen significant growth in attention to themes such as global citizenship education, global and/or intercultural competences, education for democratic citizenship in higher education programmes, particularly in the ‘global north’ (Bourne, 2020; OECD, 2018; Stein, 2020). The development of global competences and citizenship has generally been addressed through student mobility with international exchanges, volunteering and/or service learning programmes (Jackson, 2018; Streitwiser and Light, 2009) and more recently, internationalisation at home (Beelen & Jones, 2015; O’Dowd, 2022). There is often the assumption that merely having this kind of experience will automatically lead to the development of intercultural and global competences, though research shows that this is often not the case (Beaven & Borghetti, 2016; Richardson, 2016). Furthermore, there has been a growing questioning of global citizenship education, the values it is based upon, and for whose benefit (Andreotti & Souza, 2012; Stein & Andreotti, 2016).

The COVID-19 pandemic has both heightened the relevance of global citizenship education and challenged universities’ capacities for offering students global learning experiences. The pandemic highlighted our interconnectedness and interdependence, and the need for global collaboration and action (Hazelkorn & Locke, 2020). However it has also very clearly brought to light existing inequities in the world (Bozkurt et al. 2020; Milan & Masiero, 2021). As Czerniewicz et al. write from the South African context, “The current crisis has made it impossible not to recognise the historical, geospatial, economic inequalities of the country and the world students live in. In a certain sense, the pandemic, and the pivoting to online made visible, the invisible, or ignored manifestations and mechanisms of inequality.” (2020, p. 949).

Well before the current crisis, the motivations and ethics of universities’ relentless drive for internationalisation were being called into question—issues were being raised about the commodification and neoliberalization of higher education as well as the ecological sustainability of student and staff mobility (de Wit & Altbach, 2020; Stein & Andreotti, 2016). Universities have had limited success in promoting forms of global collaboration that are based on principles of reciprocity and mutual learning (Mihai, 2020) both between universities and also amongst students. Internationalization has been, for the most part, a form of neo-colonialism with universities in the ‘global north’, predominantly those in Anglophone countries, drawing in students from the ‘global south’ for the financial income they provide. Technology is also being used to reproduce relations of coloniality, with largely unidirectional flows of information from the global north to the south, for instance the MOOC phenomenon (Adam, 2019; Altbach, 2014). The recent interest in online education since the pandemic has been exacerbating this trend (Selwyn, 2023).

Universities in Europe are falling short of meeting student expectations regarding global opportunities and support, Spencer Oatey and Dauber (2019) found, and this was before the Covid-19 crisis. Their study drew on data from 2360 students, both domestic and international, responding from six universities in four European countries to the Global Education Profiler survey—a diagnostic tool that explores students’ views on the importance of various issues related to cultural diversity, including global opportunities (such as volunteering, work experience and study abroad) and support provided by institutions for understanding and fostering intercultural skills. They looked at the difference between student perspectives and their actual experiences at university and concluded that universities need to devote significant effort at providing students with global opportunities and support which can stimulate intercultural growth through engagement with difference and a better understanding of what intercultural skills are.

This article contributes to both the discussions about uses of technology for learning and for fostering global education/citizenship in higher education. This is particularly relevant at this time when universities should be reflecting on how best to use technology to enhance student learning after the emergency pivot to online learning during the Covid-19 pandemic (Selwyn, 2023). It advances current research as it provides empirical evidence on students’ transnational online experiences and what they believe they learn through these experiences as well as some of the difficulties. The paper starts with an overview of the literature first of all looking at definitions of global competences and how HEIs are fostering these competences also through virtual exchange and the evidence base supporting this practice. This is followed by a description of the context of this study, an outline of the research approach adopted and the findings. It concludes with some optimism as to how technology can potentially be harnessed to create experiences that allow for greater epistemic diversity and orientation to a more reciprocal form of internationalisation and global citizenship ‘at home’. This article also raises some questions we need to ask in order to not repeat the same mistakes as we made in the past.

## Discourses of global citizenship education

While the concept of international education is not new, this century has seen the introduction and proliferation of attention to themes such as global education (Bourne, 2020), global citizenship (Andreotti & Souza, 2012), global competency (OECD, 2018), intercultural competence, education for democratic citizenship (Council of Europe, 2010, 2017). This is also reflected in the spread of discourse regimes that develop around these inherently amorphous and ambiguous terms, which have multiple, sometimes overlapping but also often dissonant meanings and associations.

As Andreotti and Souza (2012) point out, discourses of global citizenship education are informed by different agendas and theoretical frameworks which then have different implications in terms of how they are played out in higher education. They argue that many initiatives informed by these strategies “often foreclose the complex historical, cultural and political nature of the issues, identities and perspectives embedded in global/local processes and events and in the production of knowledge about the self, the other and the world.” (2012, p. 1).

Orientations to global education/citizenship/competence are influenced by geopolitical contexts and the agendas of the organizations promoting them which may orient towards human rights and equality for all; economic competition and social mobility; social justice and intercultural understanding and/or sustainability and environmental awareness (Engel et al., 2019). The discourses of the organizations promoting global education initiatives filter down the discourse chain and influence common sense understandings and interpretations of global relations.

Most global citizenship frameworks are based on three domains of learning—the cognitive, the socio-emotional or attitudinal domain, and the behavioural. For UNESCO, Global Citizenship Education (GCED) aims “to empower learners of all ages to assume active roles, both locally and globally, in building more peaceful, tolerant, inclusive and secure societies.” (https://en.unesco.org/themes/gced/definition). UNESCO’s definition is quite broad, and refers to the need to develop specific knowledges, dispositions and values as well as behaviours. The OECD (2018) adopts the term ‘global competency’, as their focus is on the development of international measures of global competence. In 2018 in fact the OECD introduced global competence to its Programme for International Students Assessment (PISA). Their definition outlines four target dimensions of global competence which include the capacity to examine issues and situations of local, global and cultural significance (e.g. poverty, economic interdependence, migration, inequality…) and to understand different worldviews; the ability to engage positively with people from diverse backgrounds and the capacity and disposition to take constructive action toward sustainable development and collective well-being.

In discourses of the European Commission, the concept of intercultural competence has gained more currency than global competence, and the focus is generally on engagement with difference within the European region across member-states (Baumgratz, 1995) with its cultural and linguistic ‘diversity’. With reference to its European neighbours, in particular the Southern Mediterranean, the term and framework of ‘intercultural dialogue’ is preferred (De Perini, 2015). When the context becomes more global, European discourse tends to centre around citizenship and human rights education, as in the Council of Europe’s Charter on Education for Democratic Citizenship and Human Rights Education (2017).

Critics point to the tension between citizenship and competence frameworks asking whether it is possible or indeed desirable to distill broad notions of citizenship into a measurable competence framework (Joris et al., 2021). Can competence frameworks which prioritize the actions and achievements of the individual to make them more competitive in the global economy be compatible with the development of a global consciousness that is grounded in collaboration and mutual engagement with respect and acceptance of multiple ways of knowing and being?

Stein (2020) considers three approaches to global learning in Western higher education in terms of the concept of ‘global challenges’: learning about difference; learning from difference, and being taught by difference. These approaches are based on different onto-epistemological frames that dominate in Western universities.

“*Learning about difference*” is an approach that is deeply rooted within the frame of colonial modernity, within which global challenges are generally seen to be the result of insufficient knowledge, information or technology. The result is to find solutions to these ‘problems’, and to be able to do this some information about different populations and their cultures may need to be mastered by ‘the knowing subjects of Western HEIs’. Within this approach there is little to suggest engagement with different knowledge systems.

The ‘*learning from difference’* approach sees the challenge as needing to denaturalize the single story of human progress and to induce different perspectives in finding solutions and bringing positive change. As Stein puts it “Exposure to different kinds of people and knowledges is understood to generate tolerance and empathy, to in turn enable students to work together in more efficient and harmonious ways towards shared goals” (2020, p. 70). In her view, the limitation of this approach is its focus on engagements that lead to positive interpersonal relations and it avoids addressing structural relations and patterns of unequal power that can create individual discomfort and social conflict. Though many voices may be heard or shared, the scope of viable perspectives or routes is predefined.

The third approach to global learning that Stein describes is “*being taught by difference*”, which sees global challenges as the result of the imposition of the single imaginary of universal knowledges and of a shared vision for the future. Within this pedagogic response to the global challenge the limits and the damage of colonial modernity (security, certainty, supremacy, autonomy, and universality) are recognised and “radically other ways of knowing, being and relating” are encountered, without there being any attempts to control the results of the encounter. This approach requires not only cognitive engagement but also affective and relational dimensions, that is the emotional responses that arise from facing epistemic uncertainty when one’s learnt knowledges and beliefs are called into question. In Stein’s view, learning from difference entails assimilating expected and intelligible things into already existing frames of knowledge and worldviews, while being taught by difference means de-centring and being open to the unexpected and potentially disruptive rather than learning things.

## Global citizenship education in practice

Programmes to develop students’ global competences and citizenship have been growing in higher education in recent years, though there is still much work to be done (Council of Europe, 2017; Spencer Oatey and Dauber, 2019). Many of these consist of project-based learning and also ‘experiential’ programmes, such as study abroad, community service, volunteering. The latter are very much ‘embodied’ experiences which entail engagement in communities, human interactions with ‘diverse others’ (Stanlick & Szmodis, 2022).

Approaches which use online technologies have also emerged in the HEI landscape of opportunities for developing students’ global competencies (De Wit, 2016; De Wit & Altbach, 2020). These come under various forms, many of which are known as virtual exchange (Helm, 2018; O’Dowd, 2018, 2022)^1^. Virtual exchange (VE) is a pedagogic approach that uses technology and pedagogic designs to facilitate dialogue, discussion and collaboration amongst students who are situated in different geographic contexts often with the aim of fostering intercultural/global learning as well as disciplinary knowledge. VE comes in a range of shapes and sizes, with various models of virtual exchange being developed (Helm, 2018; Stevens Initiative, 2021). In the last decade large-scale funded VE programmes have been launched at national and supranational level including the U.S. Stevens Initiative which began in 2015,^2^ the European Commission’s Erasmus + Virtual Exchange^3^ pilot project which ran from 2018 to 2020 and has now become an integrated part of the Erasmus programme, Germany’s DAAD launched the IVAC programme^4^ in the wake of the pandemic and most recently the Netherlands’ Ministry of Education, Culture and Science has launched the VIS programme^5^.

There has been some diffidence towards VE, in particular as regards to the extent to which an online programme can support global citizenship and/or the development of ‘global competences’, particularly when it is seen as threatening funding for physical mobility programmes (ESU and ESN, 2021). However, there is a growing body of research which looks at the learning outcomes of different VE projects and indicates that VEs can potentially engage students in meaningful intercultural experiences in which they also develop what are seen as global competences. This evidence base ranges from case studies on specific exchanges to large scale studies which comprise multiple exchanges and hundreds, even thousands of learners.

In terms of small-scale case studies of VE that address global citizenship education, Glimäng (2022) reports on an exchange about environmental sustainability involving students located in Argentina, Poland and Sweden. She found that most groups of students tended to settle for ‘safe topics’, sharing factual information and concentrating on ‘getting the task done’ during the exchange itself. However, she found that elements of critical global awareness and the problematization of global issues underpinned by issues of power appeared in students’ individual post-exchange reflections. She suggests that project tasks might unintentionally steer learners toward safe topics and consensus but that the safe spaces that students create might be used “as a springboard to engage collaboratively with complexity” (p. 78).

Bowen et al. (2021), whose research was in the field of global health, looked at an exchange in which university students in the U.S. collaborate in teams with peers in Lebanon to address humanitarian problems in Syrian refugee camps. They found that students strongly valued learning from one another and there was mutual exchange and learning. They concluded that virtual exchanges could facilitate socially responsible global health programming.

In recent years there have been several large-scale studies on VEs which involve hundreds, even thousands of participants. The Stevens Initiative (2022) report provides data from over 3000 participants who took part in various different models of VE between the U.S. and MENA countries in two semesters, and found positive changes in many domains. They state that their data “indicate that virtual exchange participants experienced gains in global competencies over the course of the programs” (p. 10). The largest positive gains were in “knowledge of the other country or culture”. Other positive gains were reported in “perspective taking”, “self-other overlap” and “warm feelings” towards people from the other region.

In the field of teacher education, the Evaluate Group (2019) carried out a quasi-experimental research study on circa 1000 participants who had taken part in class to class VEs on themes related to education, intercultural communication and global challenges. They found slight, but steady growth in some aspects of intercultural communication, such as behavioral flexibility, interaction management and intercultural effectiveness as well as digital-pedagogical and foreign language competence in post tests in comparison to the control group. However they also found that students may tend to avoid or minimise cultural differences which they encounter in their online interactions. This minimisation of difference or ‘surfing of diversity’ has been found in other research studies on VE (O’Dowd, 2021).

## Context of this study

The Erasmus + virtual exchange (EVE) pilot project involved participants in European and Southern Mediterranean countries. The aims of this pilot project were framed in terms of intercultural dialogue, understanding of global issues and also the development of employability skills such as the ability to work in a culturally diverse team, critical thinking, understanding of global issues and the relationship between societies (Helm & van der Velden, 2020). As such the project reflected what we might call the dual nature of global citizenship projects discussed in the literature review, a neoliberal orientation towards employability and economic competitiveness of the individual and societies, as well as an orientation to intercultural understanding and social cohesion. EVE involved different typologies of virtual exchange, for instance exchanges which were co-designed by educators working in different countries, exchanges which involved participants engaging in debates on a range of issues, and dialogue-based exchanges. The final impact study compared different models of virtual exchange and found variability in the outcomes of the different models implemented. Exchanges that had a strong facilitated dialogue component (that is, at least 4 weeks of 2-h video dialogue sessions led by trained facilitators) and explicitly addressed differences across ethnic and religious divides were found to lead to greater change in pre- and post test measures of warmth towards people with different ethnic and religious backgrounds than other models of exchange. Participants also reported greater perceived improvement in the ability to listen actively and showed evidence of critical thinking, questioning assumptions and engaging with complexity (Helm & van der Velden, 2021 p. 56).

This study explores in greater depth the experience of students taking part in dialogue-based exchanges and focuses on two EVE activities that involved participants in multiple facilitated dialogue sessions. These activities were the Connect Programme^6^ which centred on Online Facilitated Dialogue, and was implemented by the NGO Soliya, and iOOC activities which combined MOOC-style content with facilitated dialogues, and were implemented by the Sharing Perspectives Foundation. Each dialogue group comprised groups of 10–12 participants from a wide range of the project countries, where possible equally divided between Europe and South Mediterranean participants. For a period between 5 and 10 weeks (depending on which specific activity they did) these participants would meet the same group of students for weekly two-hour dialogue sessions, and with the guidance of trained facilitators would explore one another’s perspectives and experiences on a wide range of topics, including gender, migration, politics, identity.^7^. The most salient ‘line of difference’ in the framing of the project was across the Mediterranean ‘border’, European countries north of the Mediterranean and countries in North Africa and the Middle East.

The participants were mainly university students who took part in this activity as an optional or compulsory part of a curricular course. There was considerable variety in how these exchanges were integrated into university curricula, for some participants it was, for example, a component of their English course, for others it could be an optional module, for example on intercultural communication, global competences.

In this study we explore participants’ discourses as they report on their main learning outcomes and what they enjoyed through their virtual exchange experience as these can provide insights into how participants make sense of their online experience. We look at how their discourses relate to the concepts of global competence or global citizenship as we have described above.

### Research questions

1. How do participants describe the learning outcomes of their virtual exchange experience?
2. How do their discourses of learning relate to conceptualisations of global competence/citizenship education?

## Method

The study reported in this paper adopted a mixed methods, convergent design by bringing together two different but complementary datasets which regard the same dialogue exchanges (see Table 1 for a summary of dataset information). The first dataset consists of short responses to two open questions about participants’ virtual exchange experience provided by 1127 participants from over 40 different countries. These responses were gathered through a post-exchange survey administered immediately after the end of the exchanges.^8^ The responses were analysed using Reinert’s method (which will be described in detail below), a statistical approach to identifying semantic groups in qualitative data. This allows us to map the ‘lexical worlds’ of a large number of participants, and thus provides a broad, bird’s eye perspective of dominant discourses on the perceived learning of students in Europe and South Mediterranean countries.

The second dataset consists of transcripts of 2 focus groups carried out with students from a large public university in Italy who participated in VE and provides a more in-depth perspective of these students’ perceived learning outcomes. The aim of the focus groups was to dig more deeply into students’ evaluation and understanding of their VE experience and to capture a more nuanced understanding of factors contributing to their learning, evidence of critical global awareness and attitudes towards difference. Convenience sampling was used, that is students were recruited from a single institution in Italy^9^ where both VE activities had been adopted and the researchers had easy access to the participants for in-person focus groups. The activity coordinators at the institution recruited volunteer focus group participants from their students who had taken part in the exchanges. Participants in focus group 1 were Master’s level students of political science and international relations and had taken part in the Cultural Encounters programme^10^ as an alternative programme to their English course. Those in focus group 2 were Bachelor’s level students majoring in languages and had taken part in the Connect Programme^11^ as a credit-bearing elective. The focus group data was coded through qualitative content analysis, through which themes and patterns were identified. Informed consent was received from all participants in both datasets.

Reinert’s (1983, 1990, 1993, 1995) method, which has been adopted in this study, is an automated approach to topic detection through the Iramuteq software, a statistical tool for content analysis of textual data from an inductive perspective (Sbalchiero & Tuzzi, 2016). Compared to other forms of content analysis its goals are more similar to qualitative content analysis as it extracts macro-topics, or semantic groups that have a similar content. It offers a multidimensional perspective that overcomes the limits of analysing frequencies alone as it creates clusters of similar words (classes) by tracing the frontiers between the particularities of each ‘lexical world’, each of which has its own coherence. It discriminates the contours of a particular referential place constituted by a specific vocabulary, which is representative of the enunciating subject (sujet-énonciateur), and its own logic. The methodology “consists of studying the laws of vocabulary distribution in a corpus. It is concerned ‘not with finding the meaning of a text, but with determining how the elements that make up the text are organized’” (Dalud-Vincent, 2011). Reinert’s algorithm splits texts into basic context units of similar length (these can be sentences, or fragments of sentences). Then, the algorithm checks the occurrence and co-occurrences of content words in each unit and reports these results in a matrix (words x units). The occurrence and co-occurrence of words in units is the basis on which it assesses similarity, which is summarized through clustering. The result is a hierarchical tree diagram (dendrogram) that groups units into classes that mirror a similar lexical context. The algorithm performs a descending classification, i.e. it detects the clusters and the factors which better represent a specific lexical world (sets of units that include words that are relevant for the same cluster).

In this study, we employ Reinert’s method to examine a corpus which consists of a group of students’ responses to two different questions related to their learning outcomes for participating in the virtual exchange projects carried out by Soliya and Sharing Perspectives Foundation (SPF). In order to create the corpus to be analyzed in a set of units, we aggregated answers to the same question and performed a separate analysis for each question. As the dataset consisted of students’ short answers, we selected “paragraphs” as a method to create text segments (TS) for the software to perform the Descending Hierarchical Analysis (DHA) and then the classification “simple on text”, which analyzed students’ short answers as the TS themselves, in order to avoid the fragmentation of students’ responses (Table 1).

**Table 1:** Summary of dataset information

Datasets	
Students’ responses	*What is the most important thing you learnt from this exchange?*
	1127 unique responses
	Soliya: 853 participants/ 14,292 words
	SPF: 274 participants/5454 words
	*Abstract of Iramuteq’s statistics*
	Number of texts: 1143
	Number of occurrences: 19,693
	Number of forms: 1692
	Number of hapax: 816 (4.14%of occurrences—48.23% of forms)
	Mean of occurrences by text: 17.23
	*What is the best thing you learnt from this exchange?*
	1122 unique responses
	Soliya: 840 participants/ 10,807 words
	SPF: 282 participants/ 5542 words
	*Abstract of Iramuteq’s statistics*
	Number of texts: 1159
	Number of occurrences: 16,190
	Number of forms: 1261
	Number of hapax: 578 (3.57% of occurrences—45.84% of forms)
	Mean of occurrences by text: 13.97
Focus groups	FG1 A face to face focus group in Italy with 7 Master’s level students, 2 of whom were international students (Brazil, Poland),
	FG2 A face to face focus group held in Italy with 5 undergraduate students of modern languages

## Analysis

In presenting the analysis we bring together the results in terms of lexical worlds which emerged from the statistical analysis using Iramuteq with key themes which emerged from the focus groups, illustrated with quotes from both datasets to provide a richer picture of the student discourses around their learning.

**Fig. 1:**
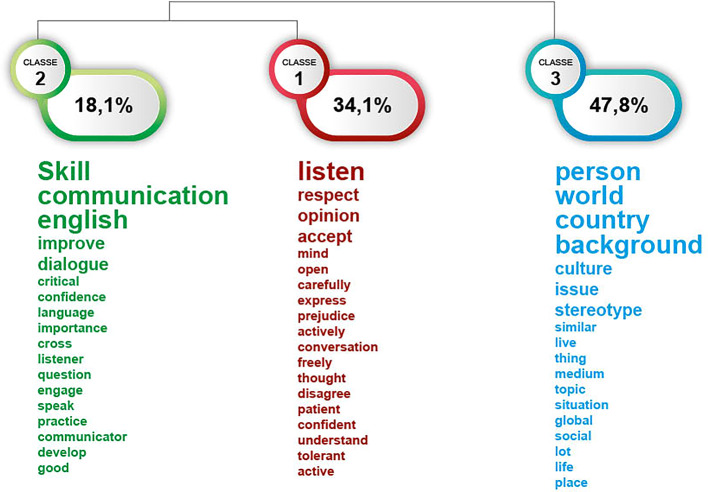
Dendogram of three lexical clusters in responses to the question “What is the most important thing you learnt from this exchange”

The Iramuteq software clusters the textual data according to the vocabulary of the corpus by employing a Descending Hierarchical Analysis (DHA). Figure 1 displays the results of the analysis as a dendrogram, which represents the clusters and their relations. The key themes which emerge from the topic detection of responses to the open question “What is the most important thing you learnt from this exchange” are expressed through three clusters, or lexical worlds.

The largest lexical world, Classe 3, comprises 47.8% of the corpus, and is linked to the concept of learning from persons who live all over the world, from different countries and backgrounds, and learning from discussing topics and issues. These keywords echo much of the mainstream discourses of international and global learning, the opportunity to encounter and learn from culturally diverse others. Below are some sample quotes from the corpus.

- That it is ok to think in a different way, and to share the way we think. that kind and nice people are out there and a change in the world is possible. (S.Med n.193)
- I learn to listen to others’ opinion, perspective and then share mine without the fear of judgment. I learned a lot about how is the situation in other part of the world that I didn’t realize before. This program helped me to open my mind even more towards differences and diversity. (Europe n.160)
- I have learned some theoretical knowledge as well as experiences of people from different countries that I couldn’t really read in a book. (Europe n.33)
- The most important thing is us getting the most benefit from each other in terms of information about the topic or about our countries and in terms of social and personal skills such as active listening, respecting perspectives even when we don’t agree with, and building friendships outside the platform. (S.Med n.219)

In the focus group discussions many of the students in both groups reported that most of their learning came from their interactions with fellow group members—and for these predominantly European students what proved most significant was the learning from their peers in the Southern Mediterranean countries, that is those who were perceived as most culturally distant. Several of the participants mentioned that prior to this experience they’d had few, if any, sustained interactions with peers from these countries, or outside of Europe. The episodes and eye opening experiences reported by focus group participants often related to their assumptions about these groups being challenged, for example one student reported mentioned the common belief that most people in South Mediterranean countries want to migrate to Europe or come here to study:

In my group there was a boy from Morocco, he wanted to know what we think about migration in Europe. We thought they wanted to come to Italy to study here but he said that he loved Morocco and never thought to travel to Italy, this challenged our stereotype that they want to travel to Europe. (FG2)

The focus group participants also remarked on how the nature of the interactions they had through the VE were quite different from other academic experiences or their everyday interactions with friends, sometimes expressing it with an element of surprise:

It is a very practical experience, you can go to conferences and summer schools, you feel you are there but you can have a more in-depth connections in a 6 weeks virtual exchange. You might know people from a long time and not discuss intercultural dialogue, because people don’t want to share this. (FG1)

The second lexical world, Classe 2, which comprises 34.1% of the corpus, is related to listening to opinions. This cluster of words contains mainly verbs: listen, respect, accept, express, disagree, understand, and adverbs such as carefully, actively and freely. These are all related to the attitudinal and behavioural domain of global citizenship education, ways of being and engaging with others. From this lexical world we get a sense of the nature of the interactions that participants were involved in, conversations in which opinions are freely expressed, people listen actively and carefully and wait for others to finish speaking.

Sample responses to the open question in the survey:

- In order to understand and actively participate, you need to listen carefully. (S. Med, n.177)
- Through my participation in this virtual_exchange i learned to be a good listener, to listen to what other mates are saying and respect their answers no matter what despite sometimes they were opposite of my own, I learned a lot about the migration topics, natives, asylum seekers…etc., and most importantly how to communicate. its was nice to share my ideas and perspectives without being shy to say them. (S.Med, n.11)
- Listening different ideas and trying to understand them i think was the most important contribution that I made (Europe, n.263)

In the analysis of the focus group discussions, listening emerged as a key theme and powerful aspect of student learning. Here there was also a strong emotional dimension in response to listening to some of the participants’ stories, for example in the extract below a participant expresses feelings of discomfort as the harsh realities of fellow participants’ daily lives and experiences of coloniality and conflict came to the fore.

Listening to people talking about political situations, how they live and so it was quite shocking for some aspects, for example the girl from Gaza—it was shocking hearing about her experience—she talked about bombs striking. There were five minutes of silence and no-one knew what to say because we were all very sad and then everyone shows [her voice wobbles] everyone shows their empathy with her. (FG2)

The smallest lexical world identified by Iramuteq relates above all to the development of what might be considered global competences, with words such ‘communication’ ‘skill’ and ‘English’ and verbs such as ‘improve’, ‘practice’, ‘develop’ and ‘speak’ and ‘dialogue’. Some sample quotes from the dataset are provided below.

- I improved my english, my communication skills. also, I learned that we shouldn’t judge people and culture without knowing them. This experience makes able to believe that communication is the key to build confidence between us. (S.Med, n.178)
- I learned collaboration and gained teamwork spirit (S.Med, n.9)
- I learned how to ask some difficult questions (Europe, n. 136)

Skill development was one of the themes also identified in the analysis of the focus group discussion. There was a duality between the participants’ focus on employability as individuals, having developed key competences for their careers through the exchange, as in the citation below, but also reflections on how what they learnt will influence them in their future in a broader sense.

It will be useful for future career, I would like to work with intercultural dialogue, and the project is focused right on this topic, teamwork is a competence you get that it is useful in your career, it is an experience which counts in your CV also when you go to a job interview and you show that you did an intercultural project, they are hiring people with more an international background. (FG1)

**Fig. 2:**
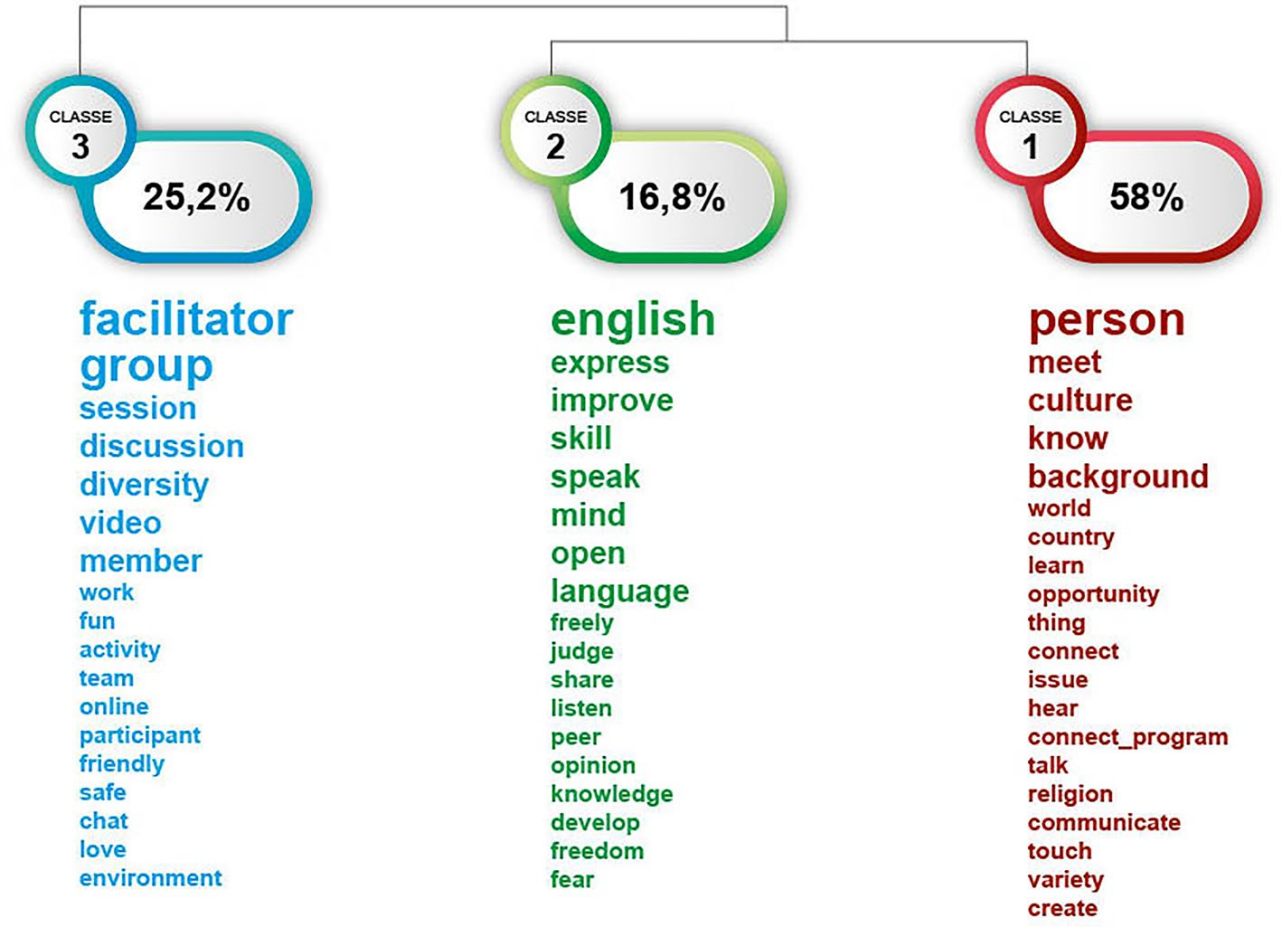
Dendogram of lexical worlds in responses to the question: what was the best thing about your exchange?

Responses to the open question asking participants what they liked best about the virtual exchange programme were also analysed and here again, there were quite distinct areas but which overlap considerably with the learning outcomes reported above. Once again the interpersonal dimension is the most prominent (see Fig. 2), ‘meeting persons’ from different countries, culture, background. Also the idea of an exchange emerges, with the prominence of the verb ‘share’ ideas, opinions and points of view. The third lexical world relates to aspects of the online ‘session’, with many words related to the various components: facilitator, group, member, participants, as well as words related to the interaction. These seem to be factors that kept participants engaged in the virtual exchange programmes.

A related theme from analysis of the focus group is appreciation for the experiential component of virtual exchange. Learning from real people, their stories, not only theory or classroom textbooks was seen as important and contributing to their learning experience.

We had international relations in practice—as you said there was a girl from Gaza—she said some parents tell their children that the bombs are fireworks—which was really sad to hear. I am sad that it is almost over. (FG1)

Although the dialogues were mediated by technology they were perceived very much as real interactions with a strong emotional impact. However the emotions reported were not only positive, almost half of the participants also talked about the discomfort they felt at times, as the quotes below show:

personally it was hard to tell them stereotypes we have in our country ...telling them what people think here was really hard—because my father, he likes Lega and Salvini so it is really hard to tell them what he says sometimes, I argue a lot with him—they supported me feeling empathy—like telling me I shouldn’t be ashamed for what we are doing and for what a lot of people think—and so telling X in Syria studying architecture who would love to travel and study abroad and he can’t because there is a war, so how can I tell him that most people can’t accept him .. but this experience also gave me the opportunity to talk about them making example—I started to tell about him. He is in Damascus. (FG2)

This latter quote suggests that this student also managed to decentre and reflect on structural inequalities and complicity. There is also evidence of the behavioural component of global citizenship in terms of taking action—in the case of this student it is by challenging others’ discourses and sharing what she learns from the experiences and knowledge gained from her peers in Syria with other people.

## Discussion

We now return to the research questions, how do participants describe the learning outcomes of VE and how do students’ discourses of learning relate to conceptualisations of global competence/citizenship education? The findings from this study show that students’ discourse highlighted above all their learning through interactions with people from different backgrounds to their own and engaging with diverse opinions on global issues and topics. Much of their engagement entailed listening, an often neglected dimension in the performance-oriented focus of global competence discourses, yet perhaps the most significant for a more critical global citizenship education. Learning to listen/hear is important in order to diminish naive understandings of the world and develop more critical readings (Freire, 2005), and according to Souza (2011) it is through learning to listen that learners come to understand their situatedness and how their values and meanings originate in the socio-historic communities they belong to.

Students also framed the learning outcomes in terms of the competences they acquired on an individual level, that is developing communication skills and being able to interact and collaborate in a diverse team with distant peers.

In terms of how the student discourses relate to conceptualisations of global citizenship education, if we refer back to Stein’s (2020) three approaches to global learning in Western higher education we see elements of both ‘learning from difference’ and ‘being taught by difference’. The dialogue-based VE provided opportunities for ‘learning from difference’ through being exposed to different kinds of people and building positive interpersonal relations, developing empathy and accepting different opinions and worldviews. To what extent though did the voices that they were exposed and their facilitated dialogues address structural relations and patterns of inequality and/or disrupt students’ knowledge systems and certainties? Some of the words and students’ comments from the focus group discussion point towards what Stein describes as “being taught by difference” and a more critical engagement with global citizenship on the part of some students. The ‘eye-opening’ stories described suggest that some of the participants were led to question their prior knowledge and were able to see its incompleteness and partiality. Several of their comments highlighted the emotional and relational dimension of their learning experience through the exchange, which comprised feelings of discomfort, particularly when becoming aware of global inequalities and their complicity in these. This is perhaps the kind of emotional response that arises when one’s certainties and innocence or ignorance have been challenged and may be indicative of a developing critical global awareness (Stein, 2020).

This study builds on and supports previous research on virtual exchange that found that this online approach to learning can provide engaging opportunities for dialogue, collaboration and engaging in forms of intercultural and global citizenship education (Glimäng, 2022; Stevens Initiative, 2022; The Evaluate Group, 2019). What this study adds to the growing body of research is a glimpse of how intentionally designed but at the same time open-ended pedagogical virtual exchange projects which focus on dialogue and listening rather than the completion of tasks and projects offer possibilities for developing critical global awareness.

However, it is important to highlight also that there are inequities inherent in any virtual exchange practice and many voices and epistemologies which are excluded (Alami et al. 2022). Access to internet and devices as well as a degree of digital literacy are also prerequisites for participation in such exchanges and the inequities in access are well documented (Milan, Treré, and Masiero, 2021) and remain a challenge in many contexts. Beyond this is also the issue of digital capital—that is students’ behaviors and ability to use the digital learning ecologies (Wimpenny et al., 2022). Furthermore, in the case of this English-mediated VE such as those in this study, only students who have some knowledge of the English language have access to the exchanges, and though students saw the exchange as improving their language skills, different levels of competence and confidence can create unequal power dynamics and exclude many participants (Helm & Acconcia, 2019). Some scholars from the global south who see VE as an inclusive and non-hegemonic form of internationalization point out that an Intercomprehension Approach could be used to allow other languages and also knowledges to become part of such exchange programmes and thus expand the people who can participate (Guimarães, & Finardi, 2019). A future consideration might be to consider how we can engage with languages other than the written and spoken, for instance visual and performative approaches to include more diverse ways of knowing.

## Footnotes

1 Many terms have been employed to describe similar activities,collaborative online international learning (COIL) (Rubin & Guth, 2022), online intercultural exchange (Lewis & O’Dowd, 2016), telecollaboration (Guth & Helm, 2010). Most recently Virtual Exchange has come to be considered an umbrella term which comprises the many different formats that have developed (see O’Dowd 2021 for a discussion of the terminology).

2 https://www.stevensinitiative.org/.

3 https://youth.europa.eu/erasmusvirtual_en.

4 https://www.daad.de/en/information-services-for-higher-education-institutions/further-information-on-daad-programmes/ivac/.

5 https://visinhetho.nl/home/about-vis/.

6 https://youth.europa.eu/erasmusvirtual/exchanges/connect-programme_en.

7 Some of the programmes were designed around specific themes and provided short videos related to the theme, for example the 5-week Gender and Media programme, the 10-week Nationalism and populism, while in other programmes dialogue sessions were more wide-ranging.

8 The open questions were just one part of a longer, quantitative survey—results of which are reported in Helm and van der Velden, (2021).

9 Italy is the country which had a large number of participants in these activities.

10 an iOOC run by Sharing Perspectives Foundation www.sharingperspectivesfoundation.org.

11 This was run by the NGO Soliya www.soliya.net.
